# Evaluating the Nuclear Reaction Optimization (NRO) Algorithm for Gene Selection in Cancer Classification

**DOI:** 10.3390/diagnostics15070927

**Published:** 2025-04-03

**Authors:** Shahad Alkamli, Hala Alshamlan

**Affiliations:** Department of Information Technology, College of Computer and Information Sciences, King Saud University, P.O. Box 51178, Riyadh 11543, Saudi Arabia; 444203620@student.ksu.edu.sa

**Keywords:** nuclear reaction optimization (NRO), gene selection, cancer classification, microarray data, bioinformatics, optimization algorithms

## Abstract

**Background/Objectives**: Cancer classification using microarray datasets presents a significant challenge due to their extremely high dimensionality. This complexity necessitates advanced optimization methods for effective gene selection. **Methods**: This study introduces and evaluates the Nuclear Reaction Optimization (NRO)—drawing inspiration from nuclear fission and fusion—for identifying informative gene subsets in six benchmark cancer microarray datasets. Employed as a standalone approach without prior dimensionality reduction, NRO was assessed using both Support Vector Machine (SVM) and k-Nearest Neighbors (k-NN). Leave-One-Out Cross-Validation (LOOCV) was used to rigorously evaluate classification accuracy and the relevance of the selected genes. **Results**: Experimental results show that NRO achieved high classification accuracy, particularly when used with SVM. In select datasets, it outperformed several state-of-the-art optimization algorithms. However, due to the absence of additional dimensionality reduction techniques, the number of selected genes remains relatively high. Comparative analysis with Harris Hawks Optimization (HHO), Artificial Bee Colony (ABC), Particle Swarm Optimization (PSO), and Firefly Algorithm (FFA) shows that while NRO delivers competitive performance, it does not consistently outperform all methods across datasets. **Conclusions**: The study concludes that NRO is a promising gene selection approach, particularly effective in certain datasets, and suggests that future work should explore hybrid models and feature reduction techniques to further enhance its accuracy and efficiency.

## 1. Introduction

Cancer remains a significant global health challenge, necessitating advancements in diagnostic and treatment methodologies [1]. The advent of microarray technology has revolutionized molecular oncology, enabling the simultaneous analysis of thousands of genes to identify potential biomarkers for various cancers. This technological leap has brought new opportunities for precision medicine [2]. Still, these high-dimensional datasets—often incorporating thousands of genes but only a limited number of patient samples—can lead to complications, including an elevated risk of overfitting, unwieldy computational demands, and interpretive complexity.

A commonly used strategy to address these issues is feature (gene) selection. The idea is to single out a smaller set of genes that captures the essence of the data, thereby enhancing model accuracy, reducing noise, and keeping the analysis computationally manageable [3]. Throughout this paper, the terms “feature” and “gene” refer to the same concept, as each feature in the dataset represents the expression level of a specific gene. Therefore, “feature selection” and “gene selection” are used interchangeably. A variety of methods have been developed for this task, including a growing list of bio-inspired optimization algorithms. Particle Swarm Optimization (PSO) [4] and Harris Hawks Optimization (HHO) [5] have demonstrated significant capabilities in high-dimensional spaces. These methods have been successfully applied to feature-selection tasks in cancer classification, achieving high performance in identifying optimal gene subsets.

This study is the first to explore the potential of the Nuclear Reaction Optimization (NRO) algorithm [6] for optimizing gene selection in cancer microarray data. Introduced in 2019 as a general optimization algorithm, NRO has been previously applied in gene selection for cancer classification using RNA Sequencing (RNA-Seq) data [7]. However, its effectiveness with microarray data remains unexamined. This research assessed the standalone capability of the NRO algorithm to select gene subsets for cancer classification across six benchmark microarray datasets.

Here, we focused solely on the inherent strengths of the NRO algorithm, foregoing additional dimensionality-reduction techniques. By dimensionality reduction, we refer to preprocessing methods—such as filtering, statistical ranking, or feature extraction—that reduce the number of genes before applying optimization algorithms. To provide a clear baseline for NRO’s performance, we intentionally excluded prior dimensionality reduction. This approach allowed us to evaluate NRO’s capability in handling raw, high-dimensional microarray data without external influence from filtering methods.

The selected gene subsets were subsequently evaluated using robust machine learning classifiers, such as Support Vector Machines (SVMs) and k-Nearest Neighbors (k-NNs), with a primary focus on classification accuracy. The outcomes of this study were intended to position the NRO algorithm as a formidable and reliable tool in bioinformatics feature selection, thus facilitating the development of more effective and interpretable diagnostic models.

The rest of this paper is organized as follows: Section 2 provides a detailed background on classification techniques and optimization algorithms, with a focus on NRO. Section 3 reviews related works in the domain of gene selection for cancer classification. Section 4 describes the materials and methods used in this study, including the datasets, preprocessing techniques, and the implementation of the NRO algorithm. Section 5 presents the results and analysis, comparing NRO’s performance with those of other optimization methods. Finally, Section 6 concludes the paper with key findings, limitations, and directions for future research.

## 2. Background

### 2.1. Classification Techniques in Cancer Diagnosis

In the field of bioinformatics, particularly in cancer classification, specific classifiers have proven to be especially effective due to their adaptability and performance with complex, high-dimensional datasets like those generated from microarray gene-expression profiles. Here, we explore two widely used classifiers: Support Vector Machine (SVM) and k-Nearest Neighbors (k-NNs), which have distinct characteristics making them suitable for cancer detection and classification.

#### 2.1.1. Support Vector Machine (SVM)

The SVM is particularly effective for microarray data because of its ability to work well in high-dimensional spaces and its robustness to overfitting with small sample sizes. It identifies an optimal hyperplane to separate data classes, maximizing classification margins, which enhances generalization [8]. The SVM has been extensively used in cancer classification tasks to distinguish between cancer types or between cancerous and non-cancerous samples based on gene-expression profiles. Its capability to manage noisy, complex data makes it a strong benchmark for evaluating gene-selection methods [9]. The SVM is highly effective in binary classification problems and can be adapted for multiclass scenarios using strategies like one-vs.-all (OVA) or one-vs.-one (OVO) [10].

#### 2.1.2. k-Nearest Neighbors (k-NNs)

The k-Nearest Neighbors (k-NNs) is a non-parametric classifier that ranks among the most straightforward and effective methods for medical diagnosis, including cancer classification. This method operates based on a simple principle: it classifies each new case by analyzing the most common class among its nearest neighbors, determined by a chosen distance metric, typically Euclidean [11]. In gene selection, the k-NNs is valuable for assessing whether the selected genes preserve meaningful data structures and class separability. Its sensitivity to local data distributions allows it to highlight the effectiveness of gene subsets in capturing disease-specific patterns in cancer datasets [12,13].

### 2.2. Nuclear Reaction Optimization (NRO)

**Figure 1:**
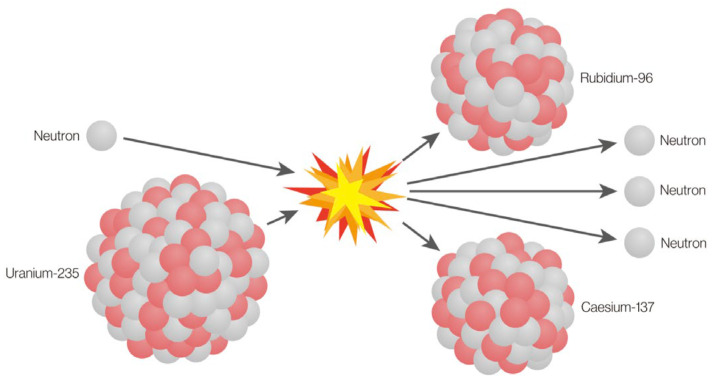
Nuclear fission process, illustrating the inspiration for solution diversification in the NRO algorithm.

**Figure 2:**
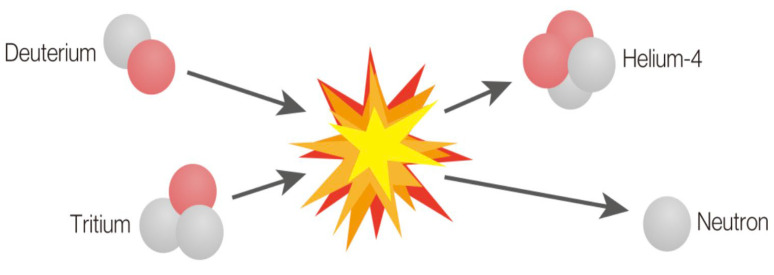
Nuclear fusion process, illustrating the inspiration for solution refinement in the NRO algorithm.

The Nuclear Reaction Optimization (NRO) algorithm is a physics-inspired metaheuristic designed to solve optimization problems by mimicking the natural processes of nuclear fission and nuclear fusion [6]. These processes simulate how nuclei split or merge to release energy. A nuclide splitting into two or more smaller nuclides is known as nuclear fission, as shown is Figure 1. The opposite is nuclear fusion. A bigger nuclide is created when two or more nuclides fuse together, as shown in Figure 2. Extremely large amounts of energy could be released by nuclear fission or fusion. In this context, it translates to exploring the solution space for optimal solutions. NRO employs a dynamic balance between exploration (searching for new potential solutions) and exploitation (refining existing solutions).

#### 2.2.1. Nuclear Fission Process

The nuclear fission process in NRO creates new candidate solutions by splitting an existing solution into smaller parts. This helps the algorithm explore different areas of the solution space and avoid becoming stuck in one region. The process uses the following equation:

(1) $$X_{i}^{Fi}\left\{\begin{array}{c} Gaussian(X_{best},\sigma_{1})+({rand}_{n}\cdot X_{best}-P_{ne}^{s}\;\cdot \;{Ne}_{i}),\;\;\;\;if\;rand\;\leq \;P_{\beta },\\ Gaussian(X_{i},\sigma_{2})+({rand}_{n}\;\cdot \;X_{best}-P_{ne}^{e}\;\cdot \;{Ne}_{i}),\;\;\;\;\;\;\;\;\;if\;rand>P_{\beta }, \end{array}\right.$$

Here,

- $X_{i}^{Fi}:\;$ the new solution generated during fission.
- $X_{best}:$ the best solution found so far.
- Gaussian(X,σ): a random value generated around X, with σ defining the spread.
- $\sigma_{1}\;,\;\sigma_{2}$ step sizes controlling the exploration range (see Equations (3) and (4) below).
- ${rand}_{n}:\;$ random number introducing variability in the solution.
- $P_{ne}^{s}\;,\;P_{ne}^{e}:\;$ mutation factors determining the scale of adjustments for subaltern and essential fission products, respectively.
- ${Ne}_{i}:$ heated neutron, calculated as ${Ne}_{i}=\frac{X_{i}}{X_{j}},$ where $X_{i}\;and\;X_{j}$ are two random solutions.
- $P_{\beta }:\;$ the probability governing whether subaltern or essential fission products are produced.

This approach ensures that some new solutions are close to the best one $X_{best}$ (local search) while others are farther away (global search), providing a good mix of exploration and refinement.

##### Step Size Adjustment in Fission

The effectiveness of the fission process depends on the step size, which determines how far new solutions deviate from existing ones. The step sizes were calculated as follows:

(2) $$\sigma_{1}=\left(\frac{\log\left(g\right)}{g}\right)\cdot \left|X_{i}-X_{\mathrm{best}}\right|,$$

(3) $$\sigma_{2}=\left(\frac{\log\left(g\right)}{g}\right)\cdot \left|X_{r}-X_{\mathrm{best}}\right|.$$

Here,

- g: current generation number; the term $\frac{\log\left(g\right)}{g}$ ensures that step sizes decrease as iterations progress.
- $\left|X_{i}-X_{\mathrm{best}}\right|:\;$ distance between the current solution and the best-known solution.
- $\left|X_{r}-X_{\mathrm{best}}\right|:$ distance between a random solution and the best-known solution.

At first, large step sizes help explore the solution space. Over time, the step sizes shrink, allowing the algorithm to refine the best solutions and reach an optimal result.

##### Mutation Factors in Fission

Mutation factors, defined as follows:

(4) $$P_{\mathrm{ne}}^{s}=\mathrm{round}\left(rand+1\right),$$

(5) $$P_{\mathrm{ne}}^{e}=\mathrm{round}\left(rand+2\right),$$

control the magnitude of adjustments applied to fission products. These factors ensure variability and are critical for balancing exploration and exploitation:

- rand:  a random number uniformly distributed between 0 and 1.
- The integer rounding ensures discrete adjustment levels for the mutation process.

These factors are incorporated into the fission equation to refine or diversify the search space, avoiding premature convergence and ensuring effective optimization.

#### 2.2.2. Nuclear Fusion Process

The nuclear fusion process refines solutions by combining promising candidates. It consists of two sub-phases: ionization and fusion.

##### Ionization Step

In this step, solutions are adjusted based on the differences between randomly selected ones. The formula is as follows:

(6) $$X_{i,d}^{Ion}\left\{\begin{array}{c} X_{r1,d}^{Fi}+rand\;\cdot \;(X_{r2,d}^{Fi}-X_{i,d}^{Fi}),\;\;if\;rand\;\leq \;0.5,\\ X_{r1,d}^{Fi}-rand\;\cdot \;(X_{r2,d}^{Fi}-X_{i,d}^{Fi})\;\;\;\;\;\;\;\;\;\;if\;rand>0.5, \end{array}\;\right.$$

Here,

- $X_{r1,d}^{Fi},\;X_{r2,d}^{Fi}$: components of two randomly selected fission solutions.
- $X_{i,d}^{Fi}:\;$ current solution.
- rand: random value for diversity.

This step prevents the algorithm from converging too early by adding diversity. If the two selected solutions are too similar, Levy flight [14] is applied to make bigger changes and avoid stagnation:

(7) $${\;X}_{i,d}^{\mathrm{Ion}}=X_{i,d}^{\mathrm{Fi}}+{\left(\alpha ⊗\mathrm{Levy}\left(\beta \right)\right)}_{d}\cdot \left(X_{i,d}^{\mathrm{Fi}}-X_{\mathrm{best},d}^{\mathrm{Fi}}\right),$$

Here,

- α: a scaling factor controlling the magnitude of jumps.
- $\mathrm{Levy}\left(\beta \right):\;$ heavy-tailed random step size, introducing both small and large adjustments.
- ⊗: indicates element-wise multiplication.
- $X_{\mathrm{best},d}^{\mathrm{Fi}}$: best-known solution in the dth  dimension.

Levy flight is specifically applied when the difference term $X_{r2,d}^{Fi}-X_{i,d}^{Fi}$ approaches zero, which could lead to stagnation in the search process. By introducing varying step sizes, this adjustment ensures that the algorithm explores new areas of the solution space, avoiding local optima.

##### Fusion Step

The fusion step combines the strengths of promising solutions to refine the search. It is expressed as follows:

(8) $$X_{i}^{\mathrm{Fu}}=X_{i}^{\mathrm{Ion}}+rand\cdot \left(X_{r1}^{\mathrm{Ion}}-X_{\mathrm{best}}\right)+rand\cdot \left(X_{r2}^{\mathrm{Ion}}-X_{\mathrm{best}}\right),$$

Here,

- $X_{i}^{\mathrm{Fu}}$: refined solution after fusion.
- $X_{\mathrm{best}}:\;$ best-known solution guiding the search.
- $X_{r1}^{\mathrm{Ion}},\;X_{r2}^{\mathrm{Ion}}:$ ionized solutions selected for comparison.
- rand: random value for diversity.

When solutions become too similar $X_{r1}^{\mathrm{Ion}}=X_{r2}^{\mathrm{Ion}}$, the algorithm may stagnate. To avoid this, Levy flight introduces random jumps, helping the algorithm explore new areas and escape local optima. This is governed by the following equation:

(9) $$X_{i}^{\mathrm{Fu}}=X_{i}^{\mathrm{Ion}}+\alpha ⊗\mathrm{Levy}\left(\beta \right)⊗\left(X_{i}^{\mathrm{Ion}}-X_{\mathrm{best}}^{\mathrm{Ion}}\right),$$

Here, Levy flight ensures that the fused solutions are not constrained to a narrow region of the search space, especially in situations in which the algorithm might otherwise fail to differentiate between nearly identical candidates.

## 3. Related Works

Gene selection for cancer classification is critical for addressing the challenges of high-dimensional microarray datasets. Numerous bio-inspired optimization algorithms have been proposed to enhance dimensionality reduction and improve classification accuracy, some of which apply dimensionality reduction before optimization [15] while others rely solely on the optimization algorithm without any prior filtering. This section reviews recent advancements in optimization-based gene-selection approaches, focusing on their methodologies and relevance to this study.

AlMazrua and AlShamlan [16] proposed Harris Hawks Optimization (HHO) for gene selection combined with SVM and k-NN classifiers to tackle the dimensionality of microarray datasets. Their approach integrated redundancy analysis and relevance scoring for preprocessing, followed by HHO-based feature selection. Evaluated on six datasets, the method achieved superior classification accuracy and reduced gene subsets compared with traditional algorithms, showcasing the efficiency of HHO in bioinformatics applications.

Alweshah et al. [17] introduced the Monarch Butterfly Optimization (MBO) algorithm as a wrapper-based feature-selection method, utilizing the k-nearest neighbor (k-NN) classifier to enhance classification accuracy and reduce computational complexity. Evaluated across 18 benchmark datasets, the MBO approach demonstrated superior performance compared with other metaheuristic algorithms, achieving an average classification accuracy of 93% while significantly reducing the number of selected features. This study highlights MBO’s effectiveness in balancing global and local search capabilities for feature-selection tasks.

Almugren and Alshamlan [18] proposed FF-SVM, a wrapper-based gene-selection algorithm combining the Firefly Algorithm (FFA) with a Support Vector Machine (SVM) classifier. The method aims to optimize cancer classification by identifying the most informative genes from high-dimensional microarray datasets. Using FFA for feature selection, followed by SVM classification with Leave-One-Out Cross-Validation, the algorithm achieved high classification accuracy with a minimal subset of genes across five benchmark datasets. Comparative experiments demonstrated the FF-SVM’s superior performance over several state-of-the-art methods in terms of accuracy and dimensionality reduction, highlighting its effectiveness in bioinformatics applications.

Nssibi et al. [19] introduced a hybrid optimization approach called iBABC-CGO, which combines an island-based Artificial Bee Colony (iABC) algorithm with Chaos Game Optimization (CGO) for gene selection. The method addresses the challenges of high-dimensional microarray datasets by using a binary representation to identify informative genes while maintaining classification accuracy. The hybrid algorithm leverages CGO principles to improve convergence and avoid local optima during the migration process, and its binary version, iBABC-CGO, ensures efficient exploration and exploitation. Experimental results on 15 biological datasets demonstrated the approach’s superior performance compared with state-of-the-art methods, highlighting its ability to achieve high accuracy with minimal feature subsets.

AlMazrua and AlShamlan [20] proposed a novel feature-selection approach for cancer classification using the Gray Wolf Optimizer (GWO). This bio-inspired optimization algorithm mimics the leadership hierarchy and cooperative hunting behavior of gray wolves to effectively explore and exploit high-dimensional datasets. Their framework utilized the GWO to identify the most significant features, achieving a balance between classification accuracy and dimensionality reduction. Their experimental results demonstrated that the method successfully reduced the number of selected features while maintaining high classification performance, highlighting its potential for bioinformatics and medical diagnostic applications.

Overall, the reviewed studies demonstrate the effectiveness of bio-inspired optimization algorithms in gene selection for cancer classification. Methods such as HHO, MBO, FFA, iBABC-CGO, and GWO have achieved high classification accuracy while selecting minimal and informative gene subsets. This study evaluates the Nuclear Reaction Optimization (NRO) algorithm as a novel contribution to further enhance gene selection and classification performance.

## 4. Materials and Methods

**Figure 3:**
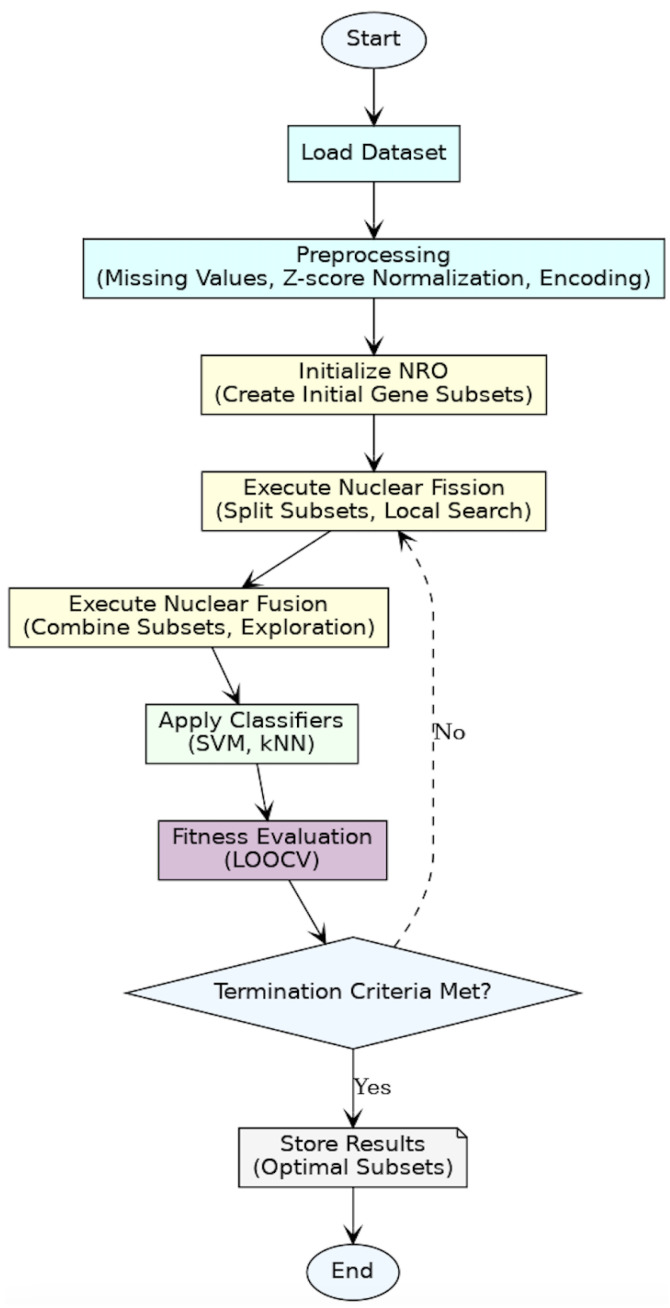
Flowchart of the methodology.

This study explores the application of the Nuclear Reaction Optimization (NRO) algorithm for gene selection in cancer classification. The methodology consists of dataset preprocessing, optimization using NRO, evaluation through machine learning classifiers, and fitness assessment. The implementation was carried out using Python (version 3.x), with numpy and pandas for preprocessing gene-expression data, scipy.io.arff for handling microarray datasets, and sklearn for feature scaling and classification. The NRO algorithm was implemented by coding its mathematical foundations, including nuclear fission and fusion processes, Lévy flight-based step size adjustments, and mutation mechanisms for optimal gene selection. Figure 3 shows the flowchart summarizing the entire methodology, from data preprocessing and optimization to fitness evaluation and termination.

### 4.1. Dataset and Preprocessing

Using six well-known binary and multiclass microarray cancer datasets that we obtained from http://www.gems-system.org/ (accessed on 15 February 2025), we assessed the overall effectiveness of the gene-selection techniques. In the discipline of bioinformatics, these datasets are frequently used to compare the effectiveness of gene-selection techniques. SRBCT [21], Lymphoma [22], and Leukemia2 [23] are multiclass microarray datasets, whereas the binary-class microarray datasets are Lung [24], Colon [25], and Leukemia1 [25,26]. We provide a thorough description of these six benchmark microarray gene-expression datasets in Table 1, including information on the number of classes, samples, and genes.

To prepare the datasets for gene selection and classification, we applied a series of preprocessing techniques commonly used in bioinformatics to ensure the datasets were clean, consistent, and suitable for machine learning algorithms. Firstly, any missing values in the datasets were handled by replacing them with the mean value of the respective gene’s expression levels using mean imputation. In our analysis, missing values were found only in the Lymphoma dataset, accounting for 4.91% of its total data, affecting 2796 genes. Given this relatively low percentage, mean imputation was applied to maintain data integrity without significantly impacting the classification performance. To standardize the features and eliminate the effects of varying scales across genes, we applied Z-score normalization, transforming the expression levels of each gene to have a mean of 0 and a standard deviation of 1. Additionally, for multiclass datasets, we employed label encoding to convert categorical class labels into a numerical format, making them compatible with classification algorithms. These preprocessing steps ensured that the datasets were optimally prepared for the subsequent feature-selection and classification phases.

### 4.2. Apply Nuclear Reaction Optimization (NRO) Algorithm

The Nuclear Reaction Optimization (NRO) algorithm was adapted in this study specifically for gene selection in cancer classification using microarray data. Microarray datasets are characterized by their high dimensionality, with thousands of genes but relatively few samples, posing challenges for classification and optimization algorithms. NRO addresses these challenges by iteratively refining subsets of genes, balancing the need for compact feature sets with high classification accuracy.

Each candidate solution in the NRO algorithm is a binary vector, where 1 indicates that a gene is selected, and 0 indicates exclusion. The algorithm initialized a population of 500 such solutions, each representing a potential subset of genes. We chose a population size of 500 to ensure a diverse set of candidate solutions, which helps in effectively exploring the high-dimensional gene space while maintaining computational efficiency. A smaller population might limit diversity and lead to premature convergence, whereas a significantly larger one would increase computational cost without substantial accuracy gains. Over a maximum of 30 generations, the population evolved through nuclear fission and fusion processes. This number of generations was selected as a balance between exploration and convergence, allowing the algorithm sufficient iterations to optimize gene selection while avoiding excessive computational overhead. In our empirical tests, increasing the generations beyond 30 provided no improvements in classification accuracy.

In the context of microarray data, the nuclear fission phase introduced diversity by splitting solutions into smaller fragments, which correspond to alternative gene subsets. Equation (1) governs this process, generating new solutions by mutating the existing ones. Step sizes, dynamically adjusted using Equations (2) and (3), control the degree of variability, enabling broad exploration in early generations to identify different sets of candidate genes. As the algorithm progresses, these step sizes decrease, focusing on the refinement of the most promising gene subsets. Mutation factors from Equations (4) and (5) further adjust the solutions, ensuring that the algorithm does not converge prematurely and continues to explore the potential of unselected genes.

The nuclear fusion phase refines gene subsets by combining promising solutions. During the ionization step, solutions are adjusted using differences between randomly selected subsets, following Equation (6). This step ensures the algorithm can evaluate combinations of genes that might not have been initially included in a single solution. For microarray data, in which many genes have weak but complementary contributions to classification, this step is crucial. When the differences between the selected solutions are small, Lévy flight from Equation (7) is applied to introduce large jumps, allowing the algorithm to escape local optima and explore new subsets of genes.

In the fusion step, refined subsets are combined using Equation (8), producing solutions that integrate information from the best-performing gene subsets of previous generations. Lévy flight, applied when necessary through Equation (9), ensures that the fusion phase continues to explore new regions of the search space rather than stagnating on similar subsets. This capability was critical for handling the complexity of microarray data, in which interactions among genes can lead to nonlinear relationships affecting the classification accuracy.

The algorithm evaluated each solution using the fitness function described in Section 4.4, which combines LOOCV-based classification accuracy with a penalty for large subsets. The process continued until the maximum number of generations was reached. The final output was an optimized subset of genes that balanced high classification accuracy with minimal dimensionality, ensuring that the selected genes were both computationally efficient and biologically informative for cancer classification. The pseudo code of the NRO algorithm is shown in Algorithm 1.
**Algorithm 1:** Nuclear Reaction Optimization (NRO) Algorithm for Gene Selection>**Require:** Population size N = 500, Maximum generations T = 30
**Ensure:** Optimized subset of gene
                           ▷ **Initialization**
1: Initialize a population of 500 binary solutions $x_{i}$, where $x_{i}$ [j] = 1 if gene
   j is selected, otherwise $x_{i}$ [j] = 0
2: Set bounds [0, 1] for solutions
3: Initialize global best solution $X_{\mathrm{best}}$
4: Compute initial fitness of $X_{\mathrm{best}}$ using LOOCV
5: **for** g = 1 to T **do**
                     ▷ **Fission Phase: Introduce Diversity**
6:     **for** each solution $x_{i}$ in the population **do**
7:       Generate new solutions as per Equation (1)
8:       Adjust step sizes dynamically as per Equations (2) and (3)
9:       Apply mutation factors as per Equations (4) and (5)
10:     **end for**
                     ▷ **Fusion Phase: Refine Solutions**
11:     **for** each solution $x_{i}$ in the population **do**
12:       Adjust solutions through ionization as per Equation (6)
13:       **if** Ionized solutions are nearly identical **then**
14:         Apply Lévy flight adjustment as per Equation (7)
15:       **end if**
16:       Combine solutions through fusion as per Equation (8)
17:       **if** Fused solutions are nearly identical **then**
18:         Apply Lévy flight adjustment as per Equation (9)
19:       **end if**
20:     **end for**
                     ▷ **Fitness Evaluation using LOOCV**
21:     **for** each solution $x_{i}$ in the population **do**
22:       Compute classification accuracy using LOOCV
23:       Compute fitness: Fitness($x_{i}$) = Accuracy *−* Penalty(size($x_{i}$))
24:       **if** $\mathrm{Fitness}(x_{i}$$)\;>\;\mathrm{Fitness}(X_{\mathrm{best}}$) **then**
25:         $\;\mathrm{Update}\;X_{\mathrm{best}}$ *←* $x_{i}$
26:       **end if**
27:     **end for**
28: **end for**
29: Return $X_{\mathrm{best}}$ Optimized subset of genes

### 4.3. Apply Classifiers

To evaluate the performance of the gene subsets selected by the NRO algorithm, two machine learning classifiers were employed: Support Vector Machine (SVM) and k-Nearest Neighbors (k-NNs). These classifiers were chosen for their established effectiveness in handling high-dimensional data, making them particularly suitable for microarray datasets with thousands of genes but relatively few samples.

The SVM was configured with a linear kernel and a penalty parameter (C = 1), which is effective for binary classification tasks in which the data are high-dimensional and sparse. Its ability to maximize the margin between classes ensures robustness in separating cancerous and non-cancerous samples, as well as in distinguishing between different cancer subtypes.

The k-NN classifier was configured with k = 5, using Euclidean distance to measure similarity between samples. Its non-parametric nature allows it to adapt well to the inherent clustering within microarray datasets, particularly when the dimensionality has been reduced through gene selection.

Both classifiers were integrated into the fitness evaluation process, in which they were used to calculate the classification accuracy through Leave-One-Out Cross-Validation (LOOCV). By applying SVM and k-NNs to the gene subsets generated by NRO, the methodology ensures that the selected subsets are rigorously evaluated for their ability to distinguish between cancer classes.

### 4.4. Fitness Evaluation

The fitness evaluation process measures the quality of each candidate gene subset by balancing classification performance with dimensionality reduction. The fitness of a solution is computed as follows:

(10) Fitness=LOOCV Accuracy−Penalty,

where Equation (10) outlines the basic computation. The classification accuracy was calculated using LOOCV (Leave-One-Out Cross-Validation), a robust cross-validation technique suited to the small sample sizes typical of microarray datasets. In LOOCV, each sample serves as the test set exactly once, while the remaining samples form the training set. This ensures that the evaluation is both comprehensive and unbiased.

The penalty term was designed to discourage the selection of excessively large gene subsets, promoting dimensionality reduction. It was defined as follows:

(11) Penalty=0.0001×Number of Selected Genes

where Equation (11) indicates how the penalty scales with both the size of the gene subset and the progress of the optimization process, ensuring that the algorithm focuses on selecting compact subsets in later generations.

During the evaluation of alternative cross-validation frameworks, both 5-fold and 10-fold methods were tested across all datasets. These approaches resulted in classification accuracies approximately 1–2% lower than those obtained using Leave-One-Out Cross-Validation (LOOCV). Although LOOCV required significantly more computational time—typically 2 to 10 times longer depending on the dataset—it consistently produced higher accuracy. Therefore, LOOCV was selected as the cross-validation method in this study. This reflects a deliberate decision to prioritize accuracy over computational efficiency, especially in the context of small sample sizes in which each data point is critical. Moreover, numerous studies in bioinformatics have demonstrated that LOOCV provides better model reliability and generalization for high-dimensional microarray data [27,28,29,30], further supporting its adoption.

## 5. Results and Analysis

This section presents the results of applying the Nuclear Reaction Optimization (NRO) algorithm for gene selection in cancer classification across six benchmark microarray datasets. The performance of NRO was evaluated in terms of the number of selected genes and classification accuracy, followed by an analysis of precision, recall, and F1-score to further assess classification robustness. Additionally, the results are compared with state-of-the-art gene-selection algorithms to assess the relative effectiveness of NRO.

The results presented in Table 2 reveal that the NRO algorithm effectively selects informative gene subsets for cancer classification, yielding high classification accuracies across multiple datasets. However, due to the absence of dimensionality-reduction or filtering techniques, the number of selected genes remains relatively high. This was expected, as no feature-elimination techniques were applied before the optimization process.

Notably, the classification accuracy achieved with SVM consistently outperformed that of k-NNs across all the datasets. This aligned with the expectation that SVM, being more robust to high-dimensional data, would be better suited for the gene-expression datasets. The best accuracy for SVM was observed in the SRBCT dataset (99.26%), followed by Lymphoma (98.81%) and Lung (95.36%). Meanwhile, k-NNs yielded its highest accuracy in the Lung dataset (97.78%), but its overall performance was less stable, particularly for high-dimensional datasets like Leukemia1 and Leukemia2, where the classification accuracy dropped significantly for lower-performing runs.

Another key observation is that the number of selected genes varies across datasets, with some requiring a larger subset for optimal classification. For example, while only 74 genes were selected in the SRBCT dataset for SVM, the Colon dataset required 494 genes for its best-performing result. This indicates that NRO was highly adaptive in its selection process, though the lack of dimensionality reduction results in relatively large gene subsets, which could be further refined in future research.

NRO’s performance varied across datasets due to differences in gene count, sample size, and data complexity. It achieved the highest accuracy on SRBCT and Lymphoma, which had moderate gene counts and yielded smaller gene subsets. Lung also performed well despite high dimensionality, likely due to its larger sample size. Leukemia1 and Leukemia2 showed more variability, reflecting the challenge of optimizing in high-dimensional, small-sample datasets. Colon, despite having fewer genes, resulted in the lowest accuracy and the largest subsets, suggesting that feature selection was more difficult, possibly due to dataset-specific complexity.

To complement the evaluation of classification accuracy, a detailed analysis of the 95% confidence intervals (CIs) offers valuable insights into the stability and reliability of the results presented in Table 2. Confidence intervals provide a statistical range within which the true average accuracy is expected to fall, offering a measure of how consistently the NRO algorithm performs across multiple runs. In this study, the CIs were remarkably narrow, with widths ranging from 0.0019 to 0.0061 and an average width of 0.0036, indicating minimal variability and high repeatability in classification outcomes. For instance, the narrowest CI was observed for the Colon dataset using k-NNs ([0.6197, 0.6216]), while the widest CI appeared in Leukemia1 with k-NNs ([0.3731, 0.3792]); even in this case, the interval remained tight and statistically sound. Additionally, all the CIs were logically bounded between 0 and 1, validating the accuracy of the calculations. These results underscore the consistency of NRO’s performance and affirm that the reported accuracies are not outliers but represent stable, repeatable outcomes. Including confidence intervals, therefore, enriches the analysis by quantifying uncertainty and reinforcing the robustness of the findings.

While accuracy provides an overall measure of classification performance, it does not fully capture the model’s ability to minimize false positives and false negatives. To further evaluate the effectiveness of NRO-selected genes, Table 3 presents the precision, recall, and F1-score, which offer a deeper understanding of classification reliability.

Precision measures the proportion of correctly predicted positive cases out of all predicted positives. A higher precision means fewer false positives, which is crucial in medical applications to avoid unnecessary treatments. As shown in Table 3, SVM achieved perfect precision (100%) in the Lung, Lymphoma, and SRBCT datasets, indicating that it classifies all positive cases correctly without false positives. In other datasets, SVM maintained high precision, such as in Leukemia1 (98.60%) and Leukemia2 (95.81%). Meanwhile, k-NNs generally had lower precision, with its best performance in Lymphoma and Lung but lower values in other datasets, such as Colon (75.70%).

Recall, or sensitivity, measures the ability of the classifier to correctly identify all actual positive cases. A high recall reduces the risk of missing cancer cases, which is critical in medical diagnostics. SVM achieved perfect recall (100%) in the Lung, Lymphoma, and SRBCT datasets, ensuring that no positive cases are overlooked. In other datasets, recall remained high, such as in Leukemia1 (98.57%) and Leukemia2 (95.72%). The k-NNs also achieved 100% recall in the Lymphoma dataset, matching SVM, but in other cases, it performed worse than SVM, such as in Leukemia2 (80.70%) and Colon (74.39%).

The F1-score is the harmonic mean of the precision and recall, providing a balanced measure of classification performance. A high F1-score indicates that the classifier effectively minimizes both false positives and false negatives. SVM achieved an F1-score of 100% in the Lung, Lymphoma, and SRBCT datasets, reflecting its strong performance in these cases. In Leukemia1 and Leukemia2, SVM also maintained high F1-scores (98.57% and 95.70%, respectively). k-NNs reached 100% in the Lymphoma dataset but struggled in others, particularly Colon (71.34%) and SRBCT (79.70%), where its lower recall affected the overall performance.

To further evaluate the performance of NRO, Table 4 compared its SVM-based classification accuracy with other gene-selection algorithms, including Harris Hawks Optimization (HHO), Artificial Bee Colony (ABC), Particle Swarm Optimization (PSO), and Firefly Algorithm (FF). This comparison focuses solely on classification accuracy, as the majority of studies on gene-selection algorithms evaluate performance based only on accuracy. Other metrics such as precision, recall, and F1-score are not included, as they are rarely reported in gene-selection research. Accuracy remains the primary benchmark for assessing the effectiveness of selected gene subsets in classification tasks.

The results show that NRO was competitive but did not consistently outperform other methods. It achieved the highest accuracy only in the SRBCT dataset (99.26%), surpassing FF (98.8%) and other algorithms. However, in all other datasets, NRO was outperformed. For example, in the Colon dataset, NRO’s accuracy (82.16%) was the lowest, while FF achieved 98.2%. In Leukemia1, NRO reached 95.53%, which was lower than HHO (97.22%) and FF (100%). In Lung, both HHO and FF achieved 100% accuracy, surpassing NRO’s 95.36%. Similarly, in Leukemia2, NRO’s 92.47% was outperformed by ABC (97.22%) and FF (97.2%). In Lymphoma, NRO achieved 98.81%, slightly below HHO (100%).

Despite not achieving the highest accuracy in all the datasets, NRO consistently delivered a strong and reliable performance across diverse cancer datasets. Its ability to outperform methods like HHO and ABC in datasets such as Leukemia2 and SRBCT, and to achieve near-perfect accuracy in Lymphoma (98.81%) and SRBCT (99.26%), demonstrates its robustness in handling high-dimensional gene-expression data. These results highlight NRO’s effectiveness and adaptability, validating its optimization approach based on nuclear reaction mechanisms. Overall, NRO proved to be a promising and competitive algorithm for gene selection in cancer classification, with the potential for further enhancement through hybrid or refined optimization strategies.

## 6. Conclusions

This study explored the application of the Nuclear Reaction Optimization (NRO) algorithm for gene selection in cancer classification using six benchmark microarray datasets. The results demonstrated that NRO effectively identified relevant gene subsets, leading to high classification accuracy, particularly when paired with SVM. Compared with state-of-the-art optimization methods, including HHO, ABC, PSO, and FF, NRO showed competitive performance, outperforming some approaches in specific datasets such as SRBCT and Leukemia2. NRO demonstrated strong adaptability in high-dimensional feature spaces. Its ability to refine solutions through nuclear fission and fusion highlights its potential as a powerful bioinformatics tool for cancer classification. However, despite its promising results, certain limitations hinder its full efficiency in gene-selection tasks.

A key limitation of this study is the large number of selected genes. Since no dimensionality-reduction techniques were applied before running NRO, this led to reduced interpretability and higher computational costs. Additionally, the Leave-One-Out Cross-Validation (LOOCV) method, while chosen for its superior accuracy, further increased computational expense due to its iterative training process. Future work should integrate filtering techniques before applying NRO to remove irrelevant genes, improving both classification accuracy and efficiency. Furthermore, hybrid optimization approaches, in which a secondary metaheuristic is used before NRO to refine the initial population, could help accelerate convergence, enhance feature-selection quality, and reduce unnecessary computations. These improvements would make the method more scalable, interpretable, and effective for larger and more complex datasets.

Additionally, this study did not assess the biological relevance of the selected genes. Future research will include pathway enrichment analysis and comparison with known cancer biomarkers to enhance the interpretability of the results. While NRO has previously been applied to RNA-Seq data, this study demonstrates its effectiveness on microarray data. Building on these findings, future studies will evaluate NRO’s scalability and performance on larger and more complex genomic datasets.

## Figures and Tables

**Table 1:** Statistics of microarray cancer datasets.

Microarray Dataset	Classes	Samples	Total Genes
Colon [25]	2	62	2000
Leukemia1 [26]	2	72	7129
Lung [24]	2	96	7129
SRBCT [21]	4	83	2308
Lymphoma [22]	3	62	4026
Leukemia2 [23]	3	72	7129

**Table 2:** Summary of accuracy results using NRO.

Dataset	Total Genes	Classifier	Selected Genes	Accuracy			CI (95%)
				Best	Average	Worst	
Colon	2000	SVM	494	82.16%	69.53%	60.74%	[0.6904, 0.6933]
		k-NNs	615	76.11%	62.57%	54.23%	[0.6197, 0.6216]
Leukemia1	7129	SVM	308	95.53%	55.90%	27.37%	[0.5341, 0.5368]
		k-NNs	171	78.84%	38.96%	10.65%	[0.3731, 0.3792]
Leukemia2	7129	SVM	198	92.47%	52.28%	24.55%	[0.5046, 0.5083]
		k-NNs	411	79.22%	37.32%	9.35%	[0.3564, 0.3625]
Lung	7129	SVM	463	95.36%	56.53%	29.11%	[0.5482, 0.5515]
		k-NNs	117	97.78%	55.33%	27.67%	[0.5395, 0.5437]
Lymphoma	4026	SVM	119	98.81%	75.25%	59.74%	[0.7437, 0.7469]
		k-NNs	72	99.28%	75.79%	59.74%	[0.7439, 0.7486]
SRBCT	2308	SVM	74	99.26%	85.89%	76.96%	[0.8535, 0.8557]
		k-NNs	259	80.54%	67.42%	57.83%	[0.6708, 0.6732]

**Table 3:** Precision, recall, and F1-score Using NRO.

Dataset	Total Genes	Classifier	Precision	Recall	F1-Score
Colon	2000	SVM	81.25%	81.46%	81.28%
		k-NNs	75.70%	74.39%	71.34%
Leukemia1	7129	SVM	98.60%	98.57%	98.57%
		k-NNs	84.23%	82.22%	80.67%
Leukemia2	7129	SVM	95.81%	95.72%	95.70%
		k-NNs	83.37%	80.70%	80.10%
Lung	7129	SVM	100%	100%	100%
		k-NNs	99.11%	99.02%	99.04%
Lymphoma	4026	SVM	100%	100%	100%
		k-NNs	100%	100%	100%
SRBCT	2308	SVM	100%	100%	100%
		k-NNs	84.05%	81.27%	79.70%

**Table 4:** Comparison of gene-selection algorithms’ accuracies with SVM classifier for six microarray datasets.

Dataset	NRO	HHO	ABC [31]	PSO [32]	PSO [33]	FF [18]
Colon	82.16%	90.32%	95.61%	85.48%	87.01%	98.2%
Leukemia1	95.53%	97.22%	93.05%	94.44%	93.06%	100%
Leukemia2	92.47%	84.72%	97.22%	-	-	97.2%
Lung	95.36%	100%	97.91%	-	-	100%
Lymphoma	98.81%	100%	96.96%	-	-	-
SRBCT	99.26%	92.77%	95.36%	-	-	98.8%

## Data Availability

The data presented in this study are available on request from the corresponding author.
